# PLAN: a web platform for automating high-throughput BLAST searches and for managing and mining results

**DOI:** 10.1186/1471-2105-8-53

**Published:** 2007-02-09

**Authors:** Ji He, Xinbin Dai, Xuechun Zhao

**Affiliations:** 1 Bioinformatics Lab, Plant Biology Division, Samuel Roberts Noble Foundation, 2510 Sam Noble Parkway, Ardmore, OK 73401, USA

## Abstract

**Background:**

BLAST searches are widely used for sequence alignment. The search results are commonly adopted for various functional and comparative genomics tasks such as annotating unknown sequences, investigating gene models and comparing two sequence sets. Advances in sequencing technologies pose challenges for high-throughput analysis of large-scale sequence data. A number of programs and hardware solutions exist for efficient BLAST searching, but there is a lack of generic software solutions for mining and personalized management of the results. Systematically reviewing the results and identifying information of interest remains tedious and time-consuming.

**Results:**

Personal BLAST Navigator (PLAN) is a versatile web platform that helps users to carry out various personalized pre- and post-BLAST tasks, including: (1) query and target sequence database management, (2) automated high-throughput BLAST searching, (3) indexing and searching of results, (4) filtering results online, (5) managing results of personal interest in favorite categories, (6) automated sequence annotation (such as NCBI NR and ontology-based annotation). PLAN integrates, by default, the Decypher hardware-based BLAST solution provided by Active Motif Inc. with a greatly improved efficiency over conventional BLAST software. BLAST results are visualized by spreadsheets and graphs and are full-text searchable. BLAST results and sequence annotations can be exported, in part or in full, in various formats including Microsoft Excel and FASTA. Sequences and BLAST results are organized in projects, the data publication levels of which are controlled by the registered project owners. In addition, all analytical functions are provided to public users without registration.

**Conclusion:**

PLAN has proved a valuable addition to the community for automated high-throughput BLAST searches, and, more importantly, for knowledge discovery, management and sharing based on sequence alignment results. The PLAN web interface is platform-independent, easily configurable and capable of comprehensive expansion, and user-intuitive. PLAN is freely available to academic users at . The source code for local deployment is provided under free license. Full support on system utilization, installation, configuration and customization are provided to academic users.

## Background

The Basic Local Alignment Search Tool (BLAST) [1] is a widely used algorithm in the bioinformatics field. There are variants of BLAST programs besides the conventional NCBI BLAST [1], such as WU-BLAST [2] and PSI-BLAST [3]. Various solutions exist for high-efficiency BLAST searches: examples include mpiBLAST [4], BeoBLAST [5] and NBLAST [6], which are based on parallel computing, and some commercial solutions such as the Decypher server [7], which utilizes dedicated accelerator hardware. For compatibility, many BLAST solutions generate search results in the same format as NCBI's BLAST (commonly referred to as *NCBI plain text format*).

Recent developments in sequencing technologies have made studies of sequence data increasingly more convenient. With the tremendous number of sequences being generated from various large-scale projects (such as genome/EST sequencing, genome-saturating mutation through insertion/deletion, etc.), studies of functional and comparative genomics are evolving from local pairwise comparisons of a few sequences to global inspections of sequence characteristics over multiple large groupings. A few examples of the typical questions raised in these studies are: What are the major functional categories of a set of sequences; within such a set, how many sequences fall into a category of interest (such as iodine metabolism)? How many conserved sequences do two libraries share, and how many are unique to each library? Given a set of insertion/deletion mutant sequences, what are their mutation sites in the genome; are these sites randomly distributed over the genome; and do they affect specific genes? To answer these routine questions, it is essential to have not only an efficient BLAST solution for conducting high-throughput searches, but also an effective approach to managing and analyzing the search results and identifying information of interest.

Surprisingly, although many high-efficiency variations of BLAST exist, generic and effective solutions for managing and analyzing large-scale BLAST search results are lacking. Because of this, downstream analysis remains a task to be solved ad hoc by different users. Previously, in order to identify a certain sequence of interest, it was necessary to search for specific terms (such as a protein description) in a multi-megabyte result file, or to sort an Excel file that contains thousands of lines. Either approach is tedious and time-consuming. We are aware of only a few software packages that partially address these needs. For example, the EST Analysis Pipeline (ESTAP) [8], written in C++, integrates automatic BLAST searches and has a web interface for viewing and searching sequence annotations. The X Genome Initiative (XGI) developed by NCGR [9], written in JAVA and Perl, is capable of handling distributed BLAST tasks and provides a web interface for searching the BLAST results. While both systems are good for large-scale EST processing, they have limited post-BLAST analysis functions except automatic annotation and keyword searching, and lack functions that are essential or much needed in many generic functional and comparative genomics studies.

We developed a platform-independent web application named Personal BLAST Navigator (PLAN) to fill this gap. PLAN is designed to carry out various generic and routine tasks in different genomic studies. In view of the variety of user needs, instead of attempting to build a system that takes care of every detail of all BLAST-based tasks, we started with some essential "common factor" functions that are believed to be most needed by the community. Currently, PLAN integrates the Decypher hardware-based BLAST solution provided by Active Motif Inc. [7] for high-throughput BLAST searches. The system manages the query sequences and BLAST results in a user-intuitive web interface. It provides a comprehensive set of functions for managing, visualizing, filtering and searching any subset of records. PLAN also provides flexible data export and publication options for data exchange with other applications. Since it is based on an open architecture that is easily customizable and expandable, advanced functions are gradually being added in response to users' requests. After more than 14 months of continuous development, PLAN has expanded to such a scale that it is now difficult to introduce all its functions in a single article. Therefore, this paper focuses on some of PLAN's essential interactive and analytical functions that are most needed by the community. Readers are encouraged to read our online user manual and frequently asked questions for a better understanding of the system.

## Implementation

### System workflow

PLAN adopts project-based data organization. A project contains a set of logically associated data, such as a sequence library to be searched against several target databases, multiple sequence libraries to be annotated using the same criteria, or two sets of sequences to be aligned to each other. A user may have multiple projects. There is no limitation on the size of the project.

**Figure 1:**
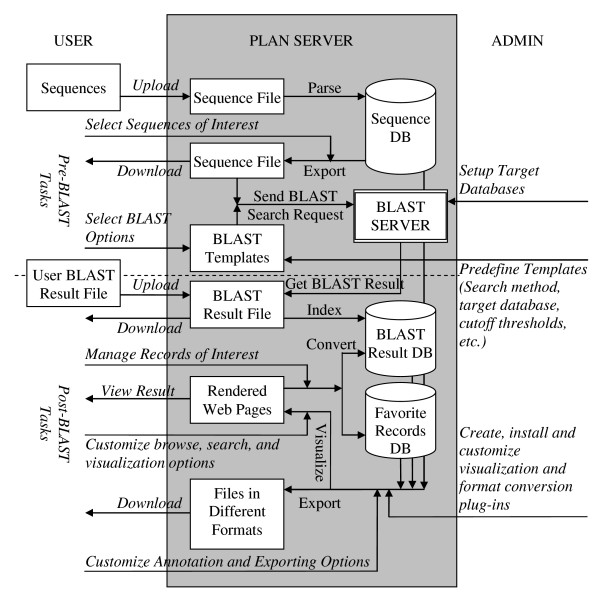
**Workflow of PLAN in each project**. Lines with *italic text *denote human-computer interactions. Lines with normal text denote server operations.

In each project, PLAN manages two types of data from users, i.e. sequence files in FASTA format and BLAST result files in NCBI plain text format. The system workflow (Figure 1) generally consists of two stages, pre-BLAST and post-BLAST. In the pre-BLAST stage, PLAN helps the user to manage sequences, customize BLAST search options and conduct BLAST searches. In the post-BLAST stage, PLAN retrieves search results from the BLAST server, imports optional custom BLAST results, manages and presents these results in different ways and performs various downstream analyses.

Query sequences are initially uploaded by users and archived in the database. Sequence ID duplication is checked and various options are provided to deal with such duplication – ignore, discard, replace, etc. BLAST searches are automated with reference to pre-defined search templates. A search template saves all necessary BLAST settings, such as alignment method (BLASTN, BLASTP, BLASTX, TBLASTX, etc.), alignment parameters (e.g. scoring matrix, score and e-value thresholds) and target database. The use of templates ensures that the user applies the same set of customized search criteria in different search sessions. The system administrator has a full control of the utilization of templates over all users, according to user grouping. For example, an institute may separate its PLAN users into public and internal groups, which can perform BLAST searches against their published or private (unpublished) sequence databases, respectively.

PLAN monitors the search process in the background and displays it online upon user request. Once the search is complete, the results, in NCBI plain text format by default, are automatically downloaded from the BLAST server and further imported into PLAN's database. The original search result files are archived for the user to download and review. A user may also upload custom search result files generated by other systems. There is no limit on the number of BLAST searches or uploaded search results in each project, and this enables the user to align multiple query sequence sets against multiple target databases for comprehensive studies.

PLAN converts text-style BLAST results into well-organized, rational database records. It indexes and hierarchically organizes the results into three levels, namely: (1) global parameters of the search session, such as search algorithm, version, database name etc.; (2) sequence level mapping between all search queries and the returned targets (hits), including bit scores and e-values (or p-values if applicable); and (3) alignment details of all high scoring pairs (HSPs). PLAN offers a wide range of high-level database manipulation functions for various post-BLAST analytical tasks. Some major post-BLAST functions are introduced in detail below.

### Multi-angle presentation of results

One of the most direct uses of PLAN is to review the BLAST results and find results of interest. PLAN adopts an intuitive web interface that simulates Microsoft Outlook. The presentation of results generally follows the three-level data hierarchy mentioned above. In brief, search session statistics are summarized in spreadsheets; sequence-level query-target mappings are displayed in spreadsheets and graphs; and various tables, texts and hyperlinks are utilized to present various alignment details. Following this schema, PLAN organizes and represents the BLAST results from several angles, summarized as follows.

**Figure 2:**
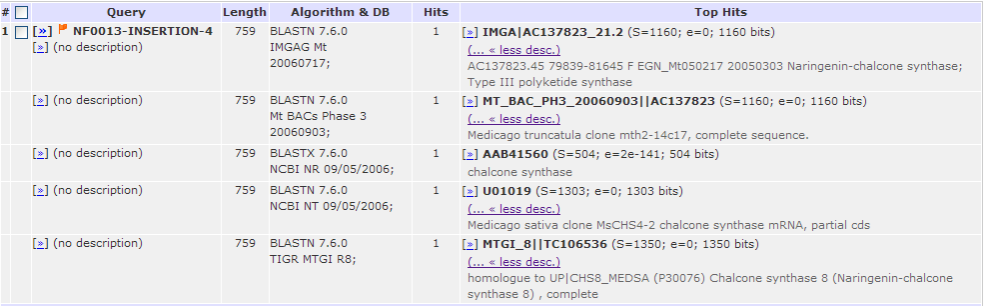
**Spreadsheet view of BLAST results according to query ID**. Multiple search results on the same query are summarized for better identification of the unknown query sequence.

First, and most intuitively, the results may be viewed according to unique query IDs. When the results are presented from this angle, PLAN summarizes and lists each query's multiple targets/hits returned from all search sessions. This facilitates comprehensive annotation of unknown sequences. Figure 2 depicts a spreadsheet for the identification of an in-house *Medicago truncatula (Mt) *insertion sequence NF0013-INSERTION-4 through searches against five major databases, namely IMGAG predicted *Mt *genes, *Mt *BAC clone genome sequences, and the NCBI protein (NR), NCBI nucleotide (NT) and TIGR *Mt *EST databases. These results not only identify the insertion site (on the basis of the result against the BAC clone database), but also suggest that the insertion potentially affects a predicted gene (implied by the result against the IMGAG database), the EST of which is included in the TIGR EST database.

Similarly, and more importantly, PLAN provides a novel, global view of BLAST results by grouping them according to unique target IDs. In other words, it helps the user to investigate which multiple queries hit the same target and how they do so. This provides useful information for many purposes. Examples include, but are not limited to, checking the redundancy of a library, estimating EST expressions *in-silico*, visualizing sequence assembly results, and investigating insertion/deletion mutation sites on the genome.

**Figure 3:**
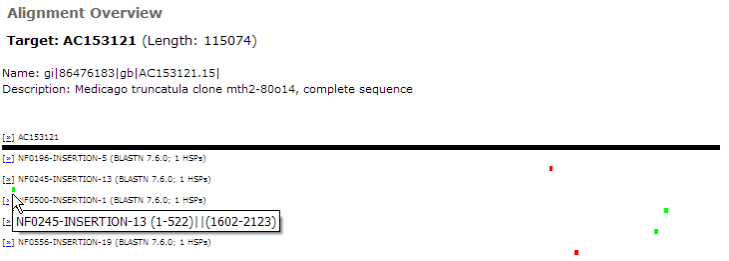
**Visualized BLAST results according to target ID**. The alignment of multiple queries (insertion sequences) on the same target (BAC clone sequence) reveals the random distribution of the insertion sites. Alignment details may be investigated by clicking on the corresponding graphical bar (Figure 4).

**Figure 4:**
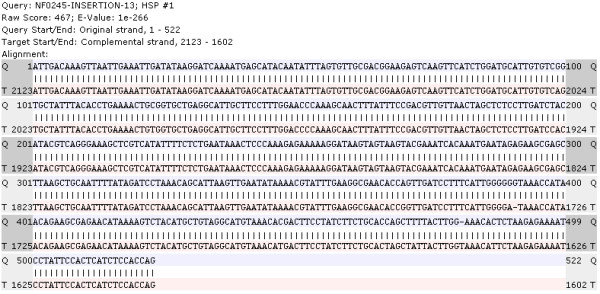
**Visualized alignment detail of a specific HSP**. The example here shows a perfect match of an insertion sequence to the BAC clone sequence.

Figure 3 illustrates how the mutation sites of five *Mt *insertion sequences are visualized on the same BAC clone sequence. These insertion sites conform to a generally random distribution. In addition, when a graphical bar (corresponding to a specific HSP) is clicked, PLAN displays the alignment details of a specific insertion sequence (Figure 4).

Besides the above intuitive data hierarchy inherited from typical BLAST results, PLAN provides a customizable plug-in mechanism, which encodes a set of translation rules provided by administrators and/or users, enabling the source of in-house sequences to be identified in terms of sequencing plate ID and gene line ID, and allowing the statistics and results to be viewed further on the basis of these in-house IDs. Such statistics are of great value in various in-house sequencing studies. Examples include, but are not limited to, checking the sequencing quality according to the percentage of "good read" sequences, validating an experiment according to data from biological replicates, and identifying unique insertion/deletion mutants in different gene lines.

Lastly, PLAN can be customized by the administrator to extract and translate certain terms in specific patterns during the presentation of results. For example, the current setup of the PLAN system on our server automatically highlights all Gene Ontology (GO) [10,11] and Plant Ontology (PO) [12,13] terms. When a GO or PO term is clicked, a window will pop up to show the corresponding ontology tree for a more in-depth study.

### Searching, filtering and organization of data of interest

PLAN provides a wide range of search options for identifying records of interest efficiently from large-scale BLAST results. It allows users to search for one or many query/target IDs, with different matching options (e.g. partial or full). It also provides a full-text search engine for matching any search term in the query/target IDs, names and descriptions. This is particularly valuable for functional analytical tasks, such as finding query sequences with hits to a specific protein family, or categorizing sequences according to functional descriptions.

In addition to these search functions, PLAN enables users to filter results on the basis of bit scores and e-value thresholds on the fly. This facilitates the review of results at different homology levels – higher bit scores and lower e-values lead to fewer results at higher homology. In addition, on each query, PLAN allows users to view a specific number of "top hits" (such as the best hit as indicated by the lowest e-value), further reducing the number of returned results for simpler yet more significant investigation. Furthermore, queries with no target (hit) may be hidden to obtain a cleaner workspace.

**Figure 5:**
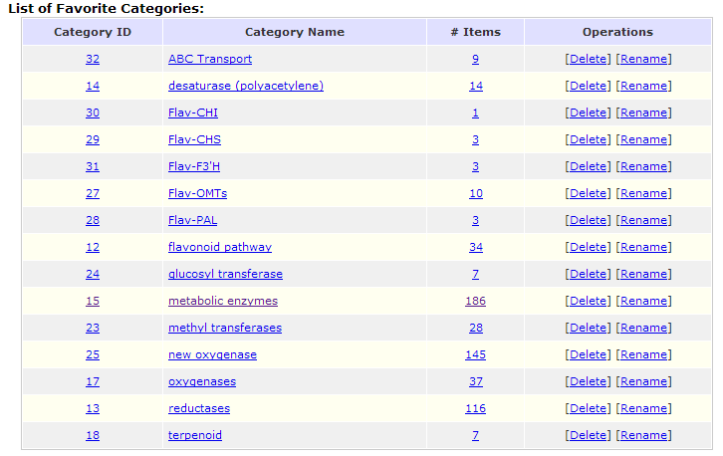
**Management of favorites**. The example here shows how results of interest are organized in different favorite categories.

It is important to have a mechanism that archives, organizes and manages records of interest for further studies. PLAN fully integrates a hierarchical "favorites" management system. A user has full control over the favorite categories (e.g. creation, edition and deletion) as well as records in each category. While the user browses any portion of the project data (such as some search results), he/she may add the data of interest to either a new or an existing favorite category. Subsequently, records in each favorite category may be browsed, removed from the category, or copied/moved to another category. Our current users consider this a very useful function. Figure 5 illustrates how PLAN assists functional genomics studies by organizing a number of sequences into different user-customized functional categories.

### Functional annotation and data export

**Figure 6:**
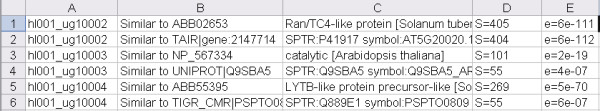
An example of multiple tab-delimited annotations of three sequences viewed in Microsoft Excel.

**Figure 7:**
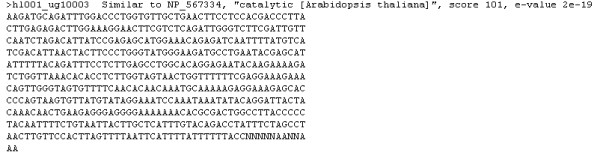
An example of natural language annotation of a sequence in FASTA format.

One of the most routine post-BLAST tasks is the functional annotation of the query sequences. PLAN provides comprehensive options for this purpose. The sequences to be annotated may be all the queries in a project, or any sequence subsets from the following sources: (1) an uploaded FASTA file, (2) a BLAST search session, (3) a favorite category, (4) an in-house gene line or plate, or (5) any user-selected sequences from any PLAN-displayed spreadsheets. A user may annotate the sequences with either of the following: (1) the top-hit in terms of either lowest e-value or highest score, or (2) all targets (hits) that passed the project filters and are visible to the user. If the query sequences have been searched against multiple databases, the user may further customize the annotation to use any one of the databases, or all of them. The output format of the annotation may be either natural language (such as "Similar to ..." or "Weakly similar to..."), which follows the format of NCBI GenBank, or tab-delimited format for downstream analysis using other software such as Microsoft Excel. PLAN may also save the user-uploaded original query sequences in well-annotated FASTA format for submission to NCBI GenBank. Figure 6 depicts the annotations of three sequences, with top hits from multiple databases, saved in tab-delimited format and viewed in Microsoft Excel. Figure 7 gives an example of a sequence being annotated with the NCBI protein database (NR) and saved in FASTA format.

In addition to these flexible annotation functions, PLAN provides multiple means of data export. Any subset of sequences and intermediate BLAST results can be exported for further analysis. For example, PLAN may export a well-formatted summary of the overlap between two libraries for Venn diagram analysis. It may also export query sequences with no hit in one search session for further searches. More information on data export may be found at the project web site.

### Control of data publication

On each project, PLAN provides three levels of data publication, summarized as follows. (1) At the most private and secure level, all data are hidden from the public. A personal password is required to access the protected data. This security level is most suitable for private, unpublished data. (2) At the medium level, data are published for read-only access. General site visitors may browse and search the project data but may not make changes. With a personal password, the project owner has a full access and all modification rights to the data. This level is best for data presentation and demonstration. (3) At the most open level, all the project data are fully accessible to all site visitors. All visitors may read and modify these data. This level is best for team work, with local deployment of the PLAN system on an institutional network. At the latter two levels, a direct hyperlink of the published project to a specific record (such as file, favorite category, gene line, plate, query or target) can be made from outside the project scope without visiting the PLAN home page. This extends PLAN as a sequence identification reference web site for knowledge sharing.

The project level is editable by the project owner at any time. For example, a user may work on his/her EST library as a private project. Once the EST library is accepted for publication, he/she may further publish the project as a read-only public project. It is worth mentioning that PLAN also provides a special category of "public projects" to generic users who want to utilize its analytical functions, without caring about data privacy or retention period. Manipulating a public project does not require user registration.

### System architecture and software implementation

**Figure 8:**
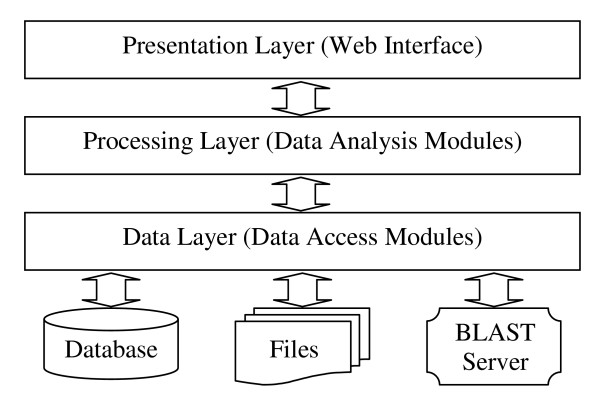
Three-Tier Architecture of PLAN.

PLAN is implemented in PHP and Perl languages. It follows a typical three-tier software architecture, comprising a presentation layer, a processing layer and a data layer (Figure 8). The presentation layer, which interacts with users, consists of a versatile web interface written in PHP. The processing layer consists of various data manipulation and analysis modules written in PHP and Perl. Some BioPHP [14] and BioPerl [15] functions are utilized for data processing. The data layer consists of a set of file handling, BLAST server communication and database-abstraction modules written in PHP and Perl. ADOdb [16] is adopted for database abstraction, which makes the system independent of the database server. The system supports, by default, the Decypher hardware-based BLAST server provided by Active Motif Inc. Software BLAST solutions may be integrated with no difficulty.

The three tiers are clearly defined while seamlessly interconnected. The platform-independent design makes PLAN capable of working on various mainstream web servers (Apache, IIS, etc.) and operation systems (Unix, Linux, Windows, etc.), and of supporting a large variety of database servers (MySQL, PostgreSQL, Oracle, Microsoft SQL, etc.). In practice, we released the system for public use on an Apache 2 web server running under the Fedora Core 5 Linux operation system, using the MySQL database and a Decypher BLAST server that contains two accelerator cards.

## Discussions and conclusion

We have developed the PLAN system for managing query sequences, automating high-throughput BLAST searches, managing, presenting and mining alignment results, and sharing personalized knowledge. Unlike the multitude of programs for different, specialized tasks, PLAN is designed to handle generic and routine analytical tasks based on BLAST searches. It is platform-independent, versatile, easy to deploy and fully customizable. It has proved a valuable addition to the community for functional and comparative genomics studies.

We have established a PLAN server for public use at our project web site. With a hardware-based BLAST solution, the server's search speed is many times higher than that of conventional BLAST software. As of September 2006, the server has been constantly online for over 10 months. It hosts over 40 projects from multiple institutes, with over 6 gigabytes of database records and over 4 gigabytes of file resources. More information about this public server, such as the user manual, data retention policy and local deployment instructions, may be found at the project web site.

The development and improvement of the system is continuous. Currently, we are implementing the automatic ontology categorization functions, starting with GO, and with PO and KEGG (Kyoto Encyclopedia of Genes and Genomes) [17] to follow. More annotation and export formats are being studied and implemented. We encourage users to submit new function requirements through our online feedback system at the project web site.

Currently, PLAN is designed as a stand-alone web platform for end users' direct use, with limited functions for data integration and sharing with other software systems. The data exchange protocols for programmable remote call and its potential integration with other systems such as a Laboratory Information Management System (LIMS) are being investigated and remain as future work.

## Availability and requirements

**Project Name: **Personal BLAST Navigator (PLAN).

**Project Home Page: **.

**Operating Systems: **Platform independent.

**Programming Language: **PHP and Perl.

**Other Requirements: **Web server with PHP support, BioPerl, and rational database server that is supported by ADOdb (MySQL server has been fully tested and is recommended). A full list of ADOdb-supported database servers may be found at .

**License: **Open-source and free for academic and non-profit use.

## Authors' contributions

JH designed, implemented the system and drafted the manuscript. XD developed part of the BLAST result parser. XZ oversaw the system design and supervised the project development. All authors read and approved the final manuscript.
